# Uncertainties in the determination of water storage changes of a shallow groundwater site using profile probe-measured volumetric water contents

**DOI:** 10.1007/s10661-025-14847-0

**Published:** 2025-12-04

**Authors:** Ottfried Dietrich, Horst H. Gerke

**Affiliations:** 1 Working Group “Lowland Hydrology and Water Management”, Research Area 2 “Land Use and Governance”, Leibniz Centre for Agricultural Landscape Research (ZALF), Eberswalder Straße 84, 15374 Müncheberg, Germany; 2 Working Group “Silicon Biogeochemistry”, Research Area 1 “Landscape Functioning”, Leibniz Centre for Agricultural Landscape Research (ZALF), Eberswalder Straße 84, 15374 Müncheberg, Germany

**Keywords:** Water storage change, Lysimeter, Volumetric water content, EnviroScan probe, Soil specific calibration

## Abstract

Water resource management of areas with shallow groundwater tables often aims at improving soil water retention and storage and at reducing greenhouse gas emissions. However, methods to quantify the effect of management measures in the field are rare. One possibility is to monitor the volumetric water content in a soil profile, to calculate the water storage change, and to upscaling the point data to the field. But how exact are the estimated values on sites with organic soils and shallow groundwater conditions? And can soil water storage changes be estimated from water content data obtained with profile probes? To test this, an EnviroScan profile probe was installed in a soil monolith of a weighable groundwater lysimeter to monitor the volumetric water contents at eight depths. The water storage changes obtained from profile probe data were compared with the reference storage change from the lysimeter. A soil specific calibration was useful especially for soil layers with higher organic matter content. Water storage changes estimated from profile probe data largely reflected the reference hydrograph of the lysimeter except for intensive drying and rapid infiltration periods. The results suggest that profile probe data can be used to quantify soil water retention; by installing multiple probes, the approach allows extending such point observation to larger lowland areas with shallow groundwater tables.

## Introduction

The improvement of water retention in lowland landscapes is a frequently discussed water management adaptation measure in Germany under changing climatic conditions. Sites with shallow groundwater tables are considered to be especially suited for the retention of water in lowlands (Bullock & Acreman, 2003; Maltby & Acreman, 2011) because they are the natural sinks for water and matter in these landscapes. These sites are located in the valleys of the last glacial period and other geomorphological depressions with high groundwater levels. Management measures to shut down their drainage and raise the groundwater levels intended to improve the water retention in the landscapes (Acreman et al., 2007; Jones et al., 2018; Stachowicz et al., 2022; Trepel & Kluge, 2002). Often, it is unclear how much water can be additionally stored by these measures. There are hardly any methods for experimentally quantifying the change in soil water storage at a site with shallow groundwater table (Jones et al., 2018). Calculations using hydrological models can estimate the water storage and the storage change based on the physical soil properties of a site, such as geometry of water bodies and changes of the water level but data that are suited for a direct validation of such model results are frequently missing (Åhlén et al., 2022). Water storage changes in lowlands with high fluctuating water tables have mostly been validated only indirectly from the water balance using discharge measurements (Blanchette et al., 2019; Evenson et al., 2018; Krasnostein & Oldham, 2004).

Many peatlands in northern Germany developed at sites with shallow groundwater conditions since the last glacial period. Most of them have been drained for an agricultural or forest use during the last 200 years so that groundwater levels dropped. This type of wetland management affected around 95% of all peatlands in Germany (Joosten et al., 2017). Only recently, a small part of the drained peatlands has been rewetted. Drained peatlands are an important source of the emissions of the greenhouse gas CO_2_ (Günther et al., 2020; Leifeld & Menichetti, 2018; Tiemeyer et al., 2016); therefore, an increase of the groundwater levels in drained peatlands can be relevant to reduce the amount of greenhouse gas emissions and plays an important role in climate protection plans of the German government (BMUV, 2021; Tanneberger et al., 2021). However, for peatland restauration to improve water retention in the landscape, there is still a lack of quantitative data on how much additional soil water could effectively be stored in such areas.

One possibility to determine the change of the soil water storage are measurements of the volumetric water content in a soil profile and their upscaling to a site. Soil moisture sensors have to be installed in different depths or profile probes can be used. Profile probes are installed with a comparatively smaller effort and provide water content values for several measurement depths (Fares & Polyakov, 2006). The amount of soil water in a soil profile can be estimated by the integration of the measured water contents over the measurement depth. The temporal change of the amount of soil water in the profile results in the relative change of the water storage. Evapotranspiration, seepage, or outflow (drainage) decrease the water storage while precipitation, inflow, or irrigation increase the water storage of a site during a time interval.

Different studies used measurements of volumetric soil water contents in soil profiles to estimate water balance components (Mounzer et al., 2008; Pachas et al., 2016; Schwartz et al., 2008; Segovia-Cardozo et al., 2022). These analyses were often designed in such a way that individual balance components could be neglected. In some studies, some water balance components were estimated in shorter periods with special boundary conditions and then these values were applied to the whole investigation period. Mounzer et al. (2008) estimated the seepage in deeper layer and concluded that the seepage was negligibly small. Therefore, the evapotranspiration could be determined as the remaining unknown of the water balance of measured water storage changes in the soil profile, precipitation, and irrigation amounts. The results were compared with the grass reference evapotranspiration and showed good agreement. Schwartz et al. (2008) estimated drainage, infiltration, and evapotranspiration from the change of soil water contents of selected time steps in an iterative way and applied them to a time series. Pachas et al. (2016) interpreted the water content changes between 6:00 p.m. and 6:00 a.m. exclusively as deep seepage of a soil profile. Therefore, they could neglect the water extraction of the plants and the evaporation during this time.

Fares and Polyakov (2006) refer to the strengths and weaknesses of the estimation of soil water balance components by means of soil water content measurements in soil profiles. Capacitive soil moisture sensors often need a soil specific calibration. This is time-consuming and labour-intensive. The measured values are affected by temperature and salinity of the soil. Sharma et al. (2021) compared several soil moisture sensors with different measurement principles and concluded that soil water storage changes based on soil moisture measurements can have larger inaccuracies. These inaccuracies are transferred to calculated water balance components, e.g. the evapotranspiration. The accuracy of water content measurements could also be affected by the soil types if default calibration functions were applied. A soil-specific calibration helped to reduce the inaccuracy (Evett et al., 2006). In a review, Evett et al. (2012) concluded that capacitive profile probes are not sufficiently precise for the estimation of the soil water balance and the evapotranspiration. Only neutron-based probes or TDR-based sensors should be used for these purposes. Nolz and Loiskandl (2017) pointed to the effect of site heterogeneity on the usefulness of measured values and their upscaling. Not all measurement locations were suited for a correct estimation of the water demand of a larger site and, for example, the control of an irrigation system. Therefore, the selection of the sensor location had a special impact on heterogeneous sites.

The listed examples from literature underline the relevance of a validation of water storage change data determined from water content measurements. A direct comparison with actual water storage changes can be performed in a small scale with weighable lysimeters, which measures the water storage change directly (Nolz & Cepuder, 2019; Sanches et al., 2019). Examples for indirect validations are comparisons with measured seepage (Vera et al., 2009) or evapotranspiration values (Segovia-Cardozo et al., 2022).

A common field of application for water content measurements with profile probes is the practical control of irrigation systems. Often, the aim was the optimisation of the control to save water and energy (Cepuder & Nolz, 2007; Fares & Alva, 2000; Miller et al., 2014; Nolz et al., 2016; Thompson et al., 2007a, 2007b; Vera et al., 2019). Thereby, the absolute values of the water storage were less important than their temporal dynamic and the requirements concerning the accuracy of the water content measurements was not as high as that for measurements of water balance components.

Shallow groundwater sites have special requirements for the quantification of soil water storage changes because the water fluxes between saturated and unsaturated zone can be directed downwards as well as upwards and plants can extract water directly from groundwater (Evett et al., 2012). Measurements of the volumetric water content in the unsaturated zone can be used to determine the soil water storage capacity of a shallow groundwater site (Nachabe et al., 2004, 2005). Nachabe et al. (2005) used this method to estimate the groundwater inflow at a shallow groundwater site by means of the storage change between 0:00 and 4:00 a.m. They assumed that the storage change was caused exclusively by inflow from groundwater during that time without evapotranspiration and continued the inflow value to the rest of the day so that the loss due to evapotranspiration could be estimated using the method as proposed by (White 1932). Still, as comparable for the application examples from sites with deep groundwater tables (see above), soil water storage changes determined in this way at sites with shallow groundwater tables also lack suitable validation.

The objective of the present study is the evaluation of the suitability of a capacitive soil moisture profile probe for the estimation of the soil water storage change on a shallow groundwater site. An EnviroScan profile probe (Sentek) with sensors in eight depths installed in a weighable groundwater lysimeter was used for the investigations. The water storage change was determined with the volumetric water contents measured in eight depths and compared with the lysimeter-measured mass change. The study aimed at evaluating the possibilities and limits of this method for application on shallow groundwater sites as a basis to improve quantification and evaluation of water retention measures in landscapes.

## Material and methods

### Groundwater lysimeter station

The field experiment was conducted in the Spreewald wetland (51° 52′ N, 14° 2′ E), around 70 km southeast of the city of Berlin. This region is characterized by an annual mean air temperature of 10.1 °C, an average annual precipitation of 566 mm, and an average annual grass reference evapotranspiration of 629 mm (1991–2020), measured at a meteorological station of the German Weather Service (DWD) in Cottbus (DWD, 2022), located 20 km south of the wetland lysimeter station.

The Spreewald wetland is part of the Spree River catchment. The wetland is located in the Baruther glacial valley of the Weichselian glacial. It is characterised by thick phreatic aquifer of around 50 m. The upper 10 m are fine and medium sands followed by medium and coarse sands in the deeper layer. A dense network of streams, channels, and ditches with a total length of 1600 km flows through it. The water level in the streams is controlled by more than 600 bigger and smaller weirs. The weirs control the distribution of water flow within the wetland as well as the inflow from and drainage to the Spree River. The grassland and forest areas in the wetland rely on recharge from the catchment to maintain the water table during the vegetation period. During wet and winter periods, the surplus water from grassland and forest areas is draining into the ditches and finally into the Spree River (Dietrich et al., 2007).

The lysimeter station is located in an area with extensive grassland agricultural, which is typical for the wetland region. The particular field is approx. 350 m wide and 1000 m long. It is bounded by ditches whose water level is controlled by small weirs. The soils show typical characteristics of peat degradation. Only the soils of depressions can be classified as peat. Locally, also clayey-humic sediment layers are present in the first metre below the soil surface. The soils in the lysimeters were classified as mollic gleysol (FAO‐ISRIC, 1990) with three horizons. This classification is based on data (Table 1) from soil samples (Dietrich & Steidl, 2021). The first horizon of the used lysimeter (0 to 40 cm depth) is a strongly degraded fen peat. It has a relatively low density (0.70 g/cm^3^) and high organic content (10.1%). The second horizon (40 to 55 cm depth) is a thin, alluvial sedimentation, typical for the Spreewald wetland, and known for its semi-permeability. It contains more clay than the other two horizons. Horizon 3 is a thick sandy soil up to the bottom of the lysimeter. The vegetation is a mix of remnants of old seed-grass stands in the drier areas and wetland plants in the wetter depressions of the area (Dietrich & Kaiser, 2017).

**Table 1:** Physical properties of the three soil layers in the lysimeter monolith (ρ_b_, bulk density; C_org_, organic carbon content; texture classes according to USDA) (Dietrich & Steidl, 2021)

Depth (cm)	Sand (%)	Silt (%)	Clay (%)	Texture	ρ_b_(g/cm^3^)	C_org_ (%)
0–40	32.8	40.5	26.6	Loam	0.70	10.1
40–55	83.1	12.8	4.1	Loamy sand	1.39	1.8
55–180	96.8	2.3	0.9	Sand	1.69	0.7

The groundwater lysimeter station consists of four weighable monoliths, each with an area of 1 m^2^ and a vertical thickness of 200 cm. The monoliths were cut in 2009 at the same place where the station is now located. The soil structure remained unchanged, and the original vegetation was preserved. Hence, the site conditions in the lysimeter represent the conditions of the surrounding area. Consequently, it can be assumed that the lysimeter largely reflects the water balance of the surrounding.

**Fig. 1:**
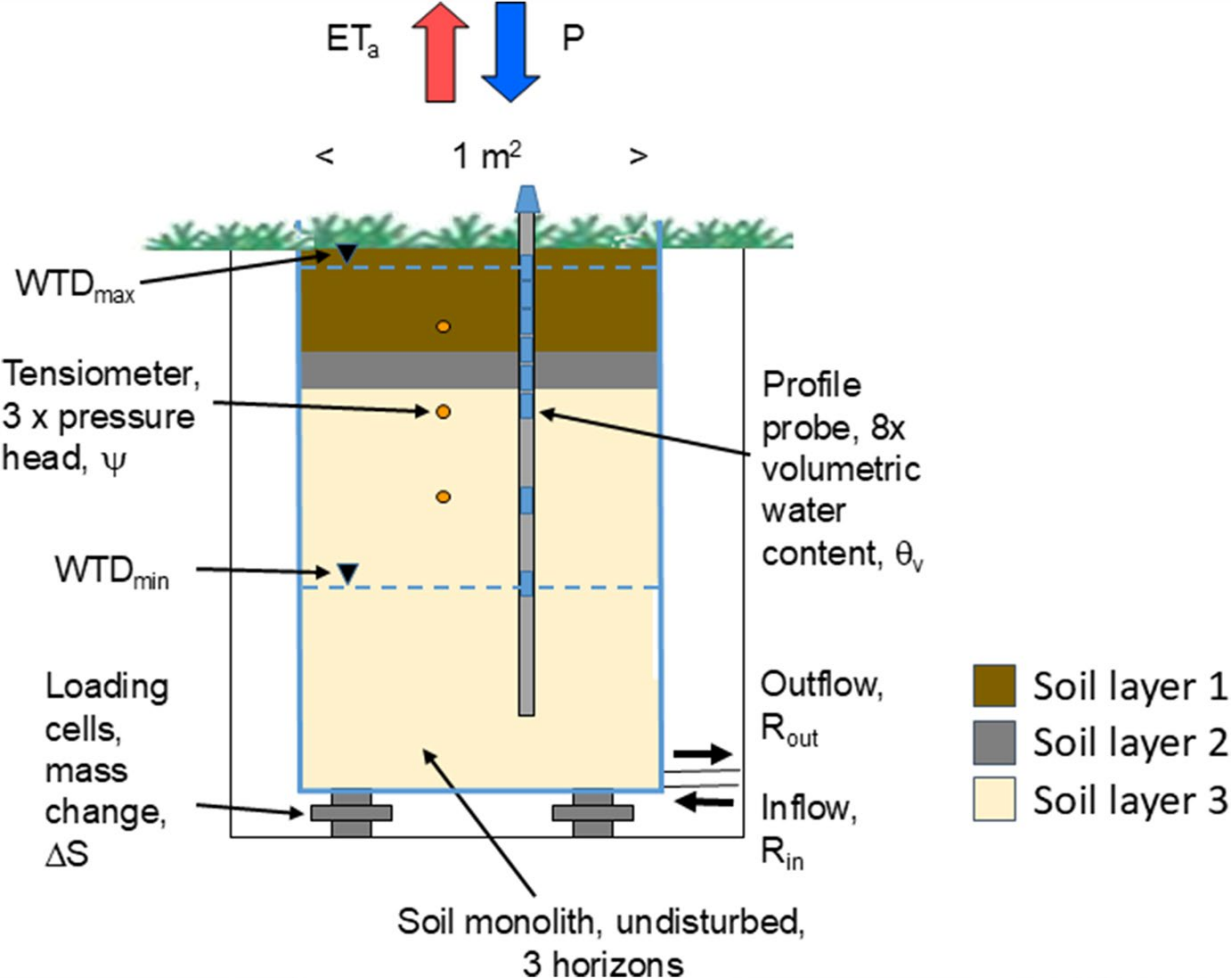
Schematic of the installed groundwater lysimeter station with monitoring parameters. WTD_max_, maximal water table depth; P, precipitation; ET_a_, actual evapotranspiration

The actual evapotranspiration (ET_a_) of the lysimeters was calculated from the water balance Eq. (1) in a post process, as:

1 $$\Delta S=P-ET_a+R_{in}-R_{out}$$

where the water balance components precipitation (P), water storage change (ΔS), groundwater inflow into the lysimeter (R_in_) and groundwater outflow out of the lysimeter (R_out_) were directly measured as illustrated in Fig. 1. Details of the lysimeter station, including sensors and controlling system of the groundwater lysimeter station, are extensively described in Dietrich et al. (2016).

The lysimeter station has been used for investigations of impacts of groundwater management strategies with different target water levels on the water balance of shallow groundwater sites since 2010. In 2018, one monolith was upgraded with a profile probe (EnviroScan, Sentek) for the measurement of the volumetric soil water content in eight depths (10 cm, 20 cm, 30 cm, 40 cm, 50 cm, 60 cm, 90 cm, and 120 cm). The sensors of the profile probe are capacitive soil moisture sensors.

An automatic weather station located close to the lysimeters determines the precipitation in 1 m height (Hellmann RG 50, Thies, 200 cm^2^); net radiation (CNR 4, Kipp & Zonen); soil heat flux (HFP01SC, Huxeflux); temperature and relative air humidity (PC-ME, Galltec + mela) in 2 m height; and the wind speed (classic, Thies) in 2.5 m height. The grass reference evapotranspiration was calculated following the method described by Allen et al. (1998).

Water balances and actual evapotranspiration were determined for hourly and daily time steps. Hourly data were only used for the calibration of the EnviroScan sensors, while all other analyses were carried out with daily values. Data from the 1st of December 2018 to 30th of November 2019 were used for the calibration of the EnviroScan sensors and the comparison of the water storage changes in the lysimeter. The time series contained a typical annual cycle of the groundwater hydrograph with higher water levels in the winter months and decreasing water levels during the vegetation period in spring and summer months.

All lysimeter data were measured in an interval of 10 min. The weather station logged data in an interval of 1 min. All data were checked for validity, and erroneous values were eliminated by post processing.

### EnviroScan calibration

Note that the EnviroScan probe uses capacitive sensors for measuring the water content in soil volume surrounding a plastic well. The minimum vertical distance between the sensors is 10 cm. Each capacitive sensor measures the frequency of a signal, which is modified by the dielectric properties of the surrounding volume. The dielectric properties depend on the soil properties and especially on the water content of the detected soil volume. The measured high frequence raw signal (F_s_) is normalised by the measured frequencies in air (F_a_) and water (F_w_) as:

2 $$SF=(F_a-F_s)/(F_a-F_w)$$

where SF is a so-called scaled frequency. The constants F_a_ and F_w_ are estimated separately for each sensor before installing the profile probe in the well. For each sensor of the profile probe, the values of SF are stored by the data logger of the lysimeter station. The volumetric water content (θ_v_) is then described with a calibration function of the type of a power function as:

3 $$\theta_v=A\ast SF^\wedge B+C$$

where A, B, and C are empirical soil specific parameters.

The EnviroScan probes are delivered and applied with a factory calibration (default). This default function (Eq. 3) has been suggested for sandy and loamy soils (Sentek, 2011) using *A* = 0.5299, *B* = 2.5775, and *C* = 0 as parameters. Several other parameters for different soil types are listed in the appendix (Table 9). The graphs in Fig. 12 illustrate the functional relation between the scaled frequency and the volumetric water content of different soil types.

For many soil types, default calibrations of soil moisture sensors were not found useful (Evett et al., 2006; Fares et al., 2004; Gabriel et al., 2010; Jabro et al., 2005; Mazahrih et al., 2008; Morgan et al., 1999; Paraskevas et al., 2012). Especially the application of capacitive sensors in soils with a high organic matter content needed a soil specific calibration (Dietrich & Steidl, 2021; Fares et al., 2016). Gravimetric or with other sensors measured soil water contents were used for the soil specific calibrations as reported for a range of soils from sandy to clayey ones (Table 9, Fig. 11, appendix). A summary of the relations between the calibration functions and the main soil types (Fig. 12, appendix) emphasizes relative similar functions for sandy soils and most of the silty soils. Many of them were similar to the default calibration function. The fewest functions are available for clayey soils. They have a big scatter around the default function. Soils with a high organic matter content like in horizon 1 of the lysimeter were not found in the literature.

Measurements with a Diviner probe were conducted on 11 dates parallel to the EnviroScan measurements. These data were used as basis for the soil specific calibration of the EnviroScan probe. The Diviner probe had a soil specific calibration for the lysimeter soils (Dietrich & Steidl, 2021). For both EnviroScan and Diviner probes, the same cased borehole (Fig. 1) was used (i.e. the same soil volume was sensed). For the Diviner measurement, the EnviroScan probe was extracted from the well for a short time, the water content was measured with the Diviner every 10 cm, and then the EnviroScan probe was mounted in the well again. Because of the different thickness of the three soil horizons, the number of measurements in each horizon differs (44 pairs of values in horizon 1, 11 pairs of values in horizon 2, 33 pairs of values in horizon 3).

The three soil horizons (Table 1) of the lysimeter soil monolith were calibrated using data from four measurement depths of the EnviroScan probe (10 cm, 20 cm, 30 cm, and 40 cm) for horizon 1, a single depth (50 cm) for horizon 2, and three depths for horizon 3 (60 cm, 90 cm, and 120 cm). In a first step, the SF values of the EnviroScan sensors were calculated (Eq. 2) using 10 min volumetric water content values based on the default calibration of the sensors. Then, in a second step, soil specific calibration functions were determined for each of the three soil layers of the lysimeter between the SF values of the EnviroScan probe and the volumetric water contents of the Diviner probe by a regression of the same type as Eq. 3.

In January 2019, there are gaps on 9 days because of problems with the data logger. These gaps were not filled.

### Water storage change

The mass of the monolith is recorded every 10 min. The data are used to determine a mass change Δm related to a reference mass m_0_ for any chosen point of time.

4 $$\Delta m=m_i-m_0=\Delta S_m$$

The mass m_i_ is the mass of the monolith at 0:00 a.m. of a day i and ΔS_m_ is actual water storage change which corresponds to Δm. Since a temporal resolution of a daily time step was used, the effects of diurnal temperature changes on the water content measurements also decreased (Baumhardt et al., 2000; Paltineanu & Starr, 1997). The monolith mass of the first day of the time series (1.12.2018) was used as the reference m_0_.

The calculation of the water storage change in a soil profile ΔS_θ_ with the measured volumetric water contents θ_v_ of the EnviroScan probe was performed in two steps. In the first step, the whole water content of the soil profile S_θi_ up to a depth of 135 cm was calculated because the groundwater level did never drop below that depth where the water storage was assumed to be constant. In a second step, the determination of the whole water storage followed in principle the approach of Nachabe et al. (2005). The eight sensors represent differently thick soil layers corresponding to their measurement depths. Simplified, this results in a depth-weighed water content S_θi_ as for up to 135 cm depth.

5 $$S_{\theta i}=\lbrack\theta_{v10}\ast15+(\theta_{v20}+\theta_{v30}+\theta_{v40}+\theta_{v50})\ast10+\theta_{v60}\ast20+(\theta_{v90}+\theta_{v120})\ast30\rbrack\ast10$$

The θ_v_ values are the measured volumetric water contents of the different depths based on the default or the soil specific calibration of the sensors in cm^3^/cm^3^. The water storages based on the default calibration of the EnviroScan probe are called “default water storage” and the water storages based on the soil specific calibrated water contents are called “soil specific water storage” in the following text. The values will be multiplied with the thickness of their corresponding depth and the factor 10. The result is the water content of the soil profile in l/m^2^ resp. mm.

The change of the water storage ΔS_θ_ related to the first value of the time series will be calculated similar to Eq. 4.

6 $$\triangle S_\theta=S_{\theta i}-S_{\theta0}$$

Again, the measured values at 0:00 a.m. of each day are used.

The comparison of the actual water storage changes with the default water storage changes resp., and the soil specific water storage changes as well as the evaluation of the quality is done by means of statistical quality criteria.

The selection of the reference values (m_0_, S_θ0_) for the determination of the water storage change (Δm, ΔS_θ_) affects some of the quality criteria (Bias, IoA, RSME, NSE). Therefore, the reference values m_0_ and S_θ0_ were optimised in a second analyse. For this purpose, the reference date was altered step by step starting with the first day of the time series and the RSME was calculated for the storage changes of both methods for each day. The date with the smallest RSME was selected as optimal reference value. Subsequently, the other quality criteria were determined for the water storage change of the weighing data ΔS_mopt_ and the water content data ΔS_θopt_.

The daily water storage changes are calculated, and their statistical values and quality criteria are determined for the lysimeter monolith (Eq. 7) and the probes (Eq. 8) as:

7 $$\Delta S_{md}=m_i-m_{i-1}$$

8 $$\triangle S_{\theta d}=S_{\theta i}-S_{\theta i-1}$$

Three groups of data were used to evaluate the effect of precipitation events on the measured daily storage changes. The first group contains all days without precipitation. The second group comprises days with a precipitation of up to 2 mm/d, and the third group consists of days with more than 2 mm/d.

### Statistics

The quality of the calibration functions was evaluated by quality criteria of the comparison between the volumetric water contents determined with the EnviroScan and Diviner probes, both with their soil specific calibration functions and the default calibration function.

Six statistical parameters (average value, median, standard deviation, minimum, maximum, range) and five quality criteria (correlation coefficient (R^2^), bias, index of agreement (IoA), root mean square error (RMSE), Nash–Sutcliffe model efficiency (NSE)) were used for the evaluation of the differently determined water storage changes. They were calculated according to the recommendations and the equations of Bennett et al. (2013) for the assessment of a model efficiency. The values based on the weight of the monolith are the reference values and the water content-based values correspond to model values.

## Results and discussion

### Sensor calibration

**Fig. 2:**
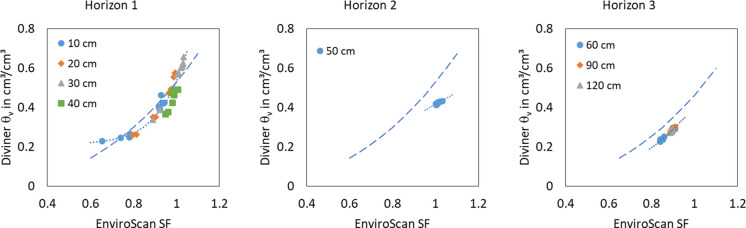
Volumetric water contents (measured with Diviner with soil specific calibration, θ_v_) and scaled frequencies of the EnviroScan sensors (SF) in different depths (symbols) with default calibration function (dashed) and soil specific calibration functions (dotted) of the three soil horizons

The measured values of the eight sensors are allocated to the three soil layers and result in three different soil specific calibration functions (Fig. 2). The soil specific functions differ from the default calibration function in slope (horizon 1), position (horizon 3), or slope and position (horizon 2). A good calibration function should cover a preferably large range of scaled frequencies and corresponding water contents. This was possible in horizon 1 with four measurement depths within the layer. The scaled frequencies range from 0.65 to 1.03 and the corresponding volumetric water contents of the Diviner probe range between 0.23 and 0.66 cm^3^/cm^3^, because there are as well as wet, medium and dry conditions with deep groundwater table depths within the data set. The results illustrate the specific requirements of organic soils (14% organic matter content) with a groundwater table near the surface for water content measurements. Water content measurements with the default calibration function would underestimate the water contents of horizon 1 for dry conditions as well as for nearly saturated conditions. Under medium soil moisture conditions, the default function overestimates the real water content (Fig. 2). The differences between both calibration functions underline the necessity of a soil specific calibration of the EnviroScan probe for horizon 1. Because of the high groundwater levels in the most time of the year (Fig. 5), this horizon has the biggest importance for the dynamic of the soil water storage in the annual cycle. Therefore, an accurate estimation of the soil water content is the precondition for the quantification of soil water storage changes at shallow groundwater sites.

All data in horizon 2 are in a relatively small range. On the one hand, this is due to the fact that there is only one measuring depth (50 cm) in this horizon. Secondly, the water content in this horizon changes only slightly under the given groundwater conditions due to the soil properties. Hence, the second horizon contributes few to the change of the water storage. Large changes in the water content of the second horizon are not reachable in a field experiment like this calibration. Only under laboratory conditions, a greater drying of the soil could be achieved. The soil specific calibration function of horizon 2 distinguishes to the default calibration function in their slope and position (Fig. 2). The default calibration overestimates the real water contents clearly.

Three measurement depths are located in horizon 3. But this horizon has saturated conditions during the most time of the year. If the groundwater levels fall below the measurement depth for short periods, the capillary rise prevents the drying of the soil in these depths. Additionally, the roots of the plants do not reach these depths so that the plants cannot extract water there. In consequence, the water contents of the sandy soil do not decrease below 0.22 cm^3^/cm^3^. Under saturated conditions, the water contents do not exceed 0.3 cm^3^/cm^3^. The measured scaled frequencies are in the range between 0.84 and 0.91. The soil specific calibration function of horizon 3 runs almost parallel to the default calibration function (Fig. 2). The measured scaled frequencies and corresponding water contents of the Diviner probe contain a relatively small range like in horizon 2. The main reason are the comparable high groundwater levels. The reason for an almost parallel course of the soil specific and default calibration functions is the soil type, which corresponds the standard soil of the default calibration much more than the soils of horizon 1 and 2.

**Fig. 3:**
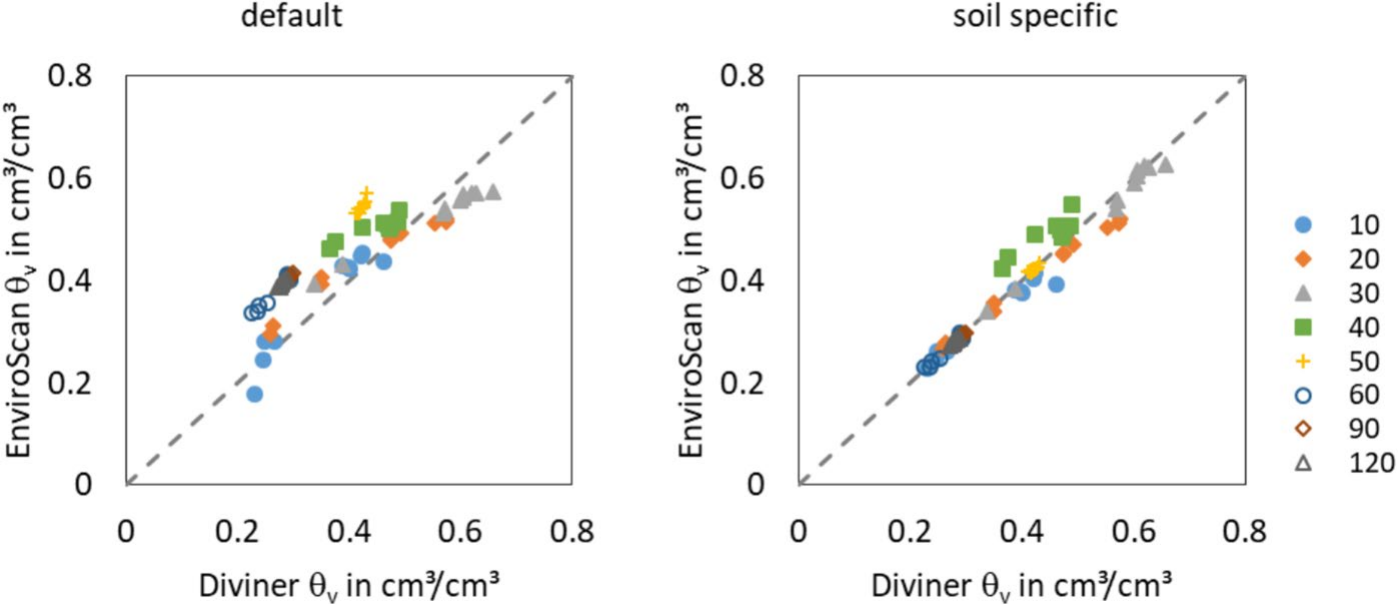
Volumetric water contents (θ_v_) measured with Diviner probe and EnviroScan probe. Diviner probe with soil specific calibration (Dietrich & Steidl, 2021). EnviroScan probe with default (left) and soil specific calibration (right), depths in legend in cm

The quality criteria of all three horizons show an improved adaptation of the volumetric water content by the soil specific calibration compared to the default calibration (Table 2). The RSME could be reduced distinctly and all NSE present a very good, clearly improved adaptation. In Fig. 3, the EnviroScan values with default calibration overestimate the Diviner values slightly. With the soil specific calibration, the agreement between both values improved (Fig. 3, right).

**Table 2:** Parameters of the default and soil specific calibration functions (A, B, and C) and quality criteria of the adaptation of EnviroScan water content to the Diviner values (correlation coefficient (*R*^2^), bias, index of agreement (IoA), root mean square error (RMSE), Nash–Sutcliffe model efficiency (NSE))

	**Default calibration**			**Soil specific calibration**		
	Horizon 1	Horizon 2	Horizon 3	Horizon 1	Horizon 2	Horizon 3
**A**	0.5274	0.5274	0.5274	0.3146	0.4148	0.4081
**B**	2.5525	2.5525	2.5525	8.1028	1.4587	3.2850
**C**	0.0000	0.0000	0.0000	0.2182	0.0000	0.0000
**R**^**2**^	0.8599	0.7730	0.9658	0.9242	0.7756	0.9658
**IoA**	0.9977	0.9841	0.9718	0.9990	1.0000	1.0000
**Bias in cm**^**3**^**/cm**^**3**^	−0.0106	−0.1216	−0.1134	0.0000	0.0000	0.0000
**RMSE in cm**^**3**^**/cm**^**3**^	0.0467	0.1218	0.1135	0.0322	0.0028	0.0034
**NSE**	0.8404	−418.23	−37.181	0.9242	0.7756	0.9658

The soil specific calibration functions indicate some more peculiarities of the soils of the study site. The scaled frequencies of the EnviroScan sensors reach values > 1 in horizons 1 and 2. Mazahrih et al. (2008) also measured scaled frequency values > 1 and referred it to the high electric conductivity of their soils. In the current case, the electric conductivity is probably affected by the high organic matter content of the soil. Another reason for measurement inaccuracies can be the allocation of the measurement depths to the soil horizons. The measurement values in 40 cm depth are allocated to horizon 1. The water contents of this sensor with the soil specific calibration function have a small deviation from the 1:1 line in Fig. 3 (right). Probably, the measurement volume of the sensor, which cover around 10 cm outside the well (Paltineanu & Starr, 1997), contains parts of the second horizon. Therefore, the soil specific calibration function describes the correlation between scaled frequency and volumetric water content for this measurement depth not perfectly. Our results underline the need for soil-specific calibration for soils with properties that have a particularly strong influence on the electromagnetic properties in the observed volume. Such properties include the organic matter content and the salt content of the soil.

### Meteorological conditions and groundwater levels

The meteorological conditions of the study period from the 1st of December 2018 to 30th of November 2019 were a little bit drier as the 30-year average conditions. The sum of the precipitation was 500 mm, and the sum of the FAO grass reference evapotranspiration was 641 mm. The deficit of the climatic water budget was −141 mm. The average values of the next weather station of the German Weather Service are 566 mm (precipitation), 629 mm (reference evapotranspiration), and −75 mm (climatic water budget) (1991/2020, Dietrich et al. (2021)). Additionally, the previous year 2018 was an extreme dry year with 325 mm precipitation and 787 mm grass reference evapotranspiration measured at the lysimeter station. It meant a deficit of −462 mm in the climatic water budget.

**Fig. 4:**
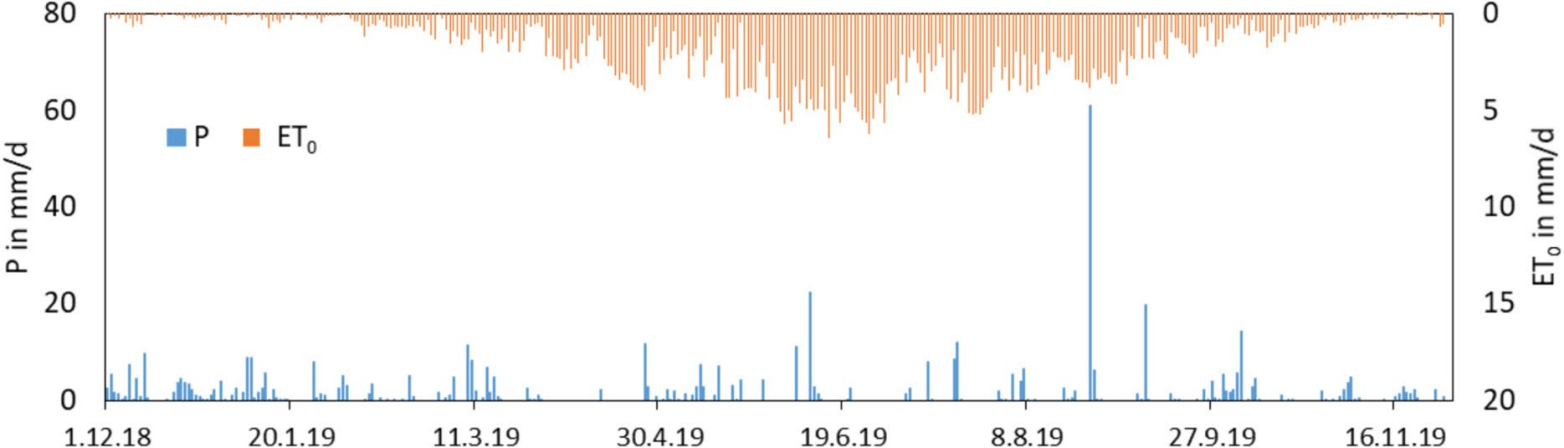
Daily sums of precipitation (P) and grass reference evapotranspiration (ET_0_) between the 1st of December 2018 and 30th of November 2019

**Fig. 5:**
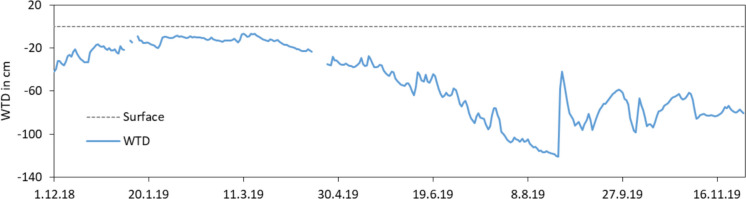
Groundwater hydrograph of the lysimeter between the 1st of December 2018 and 30th of November 2019 (measured water table depth (WTD) at 0:00 a.m.)

The precipitation events were relative uniformly distributed during the study period (Fig. 4). There was a longer period without precipitation in April with only 2 mm in 27 days. In this time, the actual evapotranspiration was not very high, and the soil was saturated up to the soil surface. Therefore, the groundwater level dropped only a few centimetres (Fig. 5). The decreasing trend continued up to end of August. The deepest water table depth was reached on the 24th of August with 124 cm below surface. On 25th and 26th of August, 67 mm precipitation caused an increase of the water level of 80 cm up to 42 cm below surface. The long period with decreasing groundwater levels were suspended by small, short increases of the water level after single precipitation events. After the heavy rainfall event end of August, the water levels oscillated between 60 and 80 cm below surface and did not reach the start level of 40 cm below surface.

### Volumetric water content measurements

**Fig. 6:**
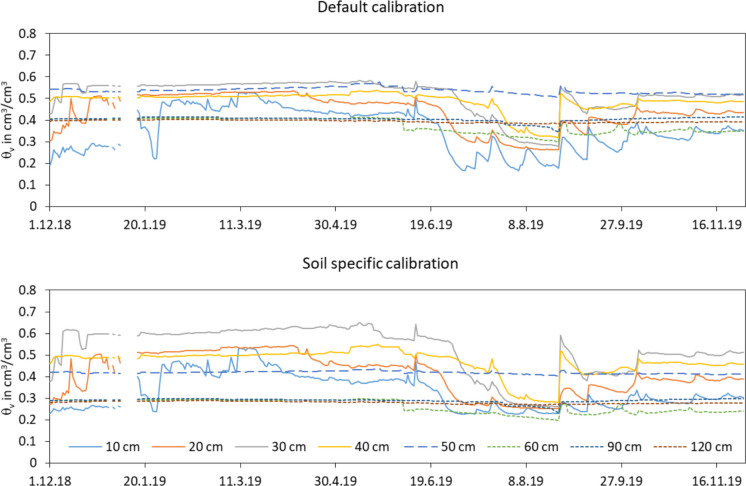
Volumetric water content measured with the EnviroScan probe in 8 depths between the 1st of December 2018 and 30th of November 2019 (daily values, top: default calibration, bottom: soil specific calibration, horizon 1: depths 10 to 40 cm, horizon 2: depth 50 cm, horizon 3: depths 60 to 120 cm)

The measured volumetric water content with default and soil specific calibration distinguishes for all three horizons (Fig. 6). The absolute values of the water content are affected by the soil types. The hydrographs of the water contents in the different depths mainly reflect the hydrograph of the water table depth and the water extraction by the plant roots.

The degraded peat of horizon 1 has volumetric water contents between 0.5 and 0.6 cm^3^/cm^3^ in the saturated state. The values can drop down to 0.24 cm^3^/cm^3^ (soil specific calibration) with decreasing groundwater levels, whereas the decrease of the water contents of the different measurement depths proceeds graded in time from top to bottom, following the drying out of the soil. The values are in the range of water contents Liu et al. (2022) explained for degraded peat soils in Germany. The water contents of all depths in horizon 1 react directly to precipitation with a fast, short increase and a following slow decrease delayed in time. The values are slightly higher under saturated conditions and slightly deeper under dry conditions with the soil specific calibration compared to the default calibration. This results in a larger difference within the annual hydrograph and increases the estimated water storage change in horizon 1.

The volumetric water content of horizon 2 (measurement depth 50 cm) is relatively constant over the whole time, but at a different level with the different calibration functions. The default calibration estimates around 0.5 cm^3^/cm^3^ and the soil specific calibration estimates around 0.4 cm^3^/cm^3^. The hydrograph of the water content does not oscillate for both calibrations. The characteristics of the soil are the reason for this special behaviour. Therefore, it is clear, horizon 2 cannot really contribute to the water storage change in the whole soil profile.

The hydrographs of the water contents in the third horizon are also relatively constant during the study period. The values with the soil specific calibration function are around 0.1 cm^3^/cm^3^ deeper as the values with the default calibration (default, 0.3 to 0.4 cm^3^/cm^3^; soil specific, 0.2 to 0.3 cm^3^/cm^3^, Fig. 6). The measured values in 60 cm depth decrease a little bit when the water table depth falls below 70 cm (starting 6th June 2019, Fig. 5). They reach their saturated values again for a short time after the heavy rainfall end of August. In 90 cm depth, the water content drops down below the saturated values for a very short time only when the water table depth falls below 110 cm. After the heavy rainfall end of August, the soil is fully saturated. In 120 cm depth, the water contents are constant, which reflects that the soil is saturated during the whole period.

The differences of the volumetric water contents between the three horizons reflect the different soil properties of the horizons. The water contents in the degraded peat layer reach values up to 0.6 cm^3^/cm^3^. In the loamy second horizon, the values are around 0.4 cm^3^/cm^3^, and in the sandy third horizon, the values do not exceed 0.3 cm^3^/cm^3^. This corresponds to the expectations for the respective soils. In dependence on the soil horizon, the water contents have another hydrograph, determined by the groundwater hydrograph, the rainfall events and water extraction of the plant roots. The hydrographs show that the first horizon contributes most to the water storage change of the whole soil profile of the three horizons. The soil of horizon 2 changes its water content only minimally over the year, as the plant roots apparently find it difficult to penetrate this soil or it is easier for the plants to extract water from the upper horizon and drain it heavily than to extract water from the soil of horizon 2. The water contents in horizon 3 change only in short periods, when the groundwater levels fall deep enough. Hence, this horizon contributes also few to the water storage change of the whole soil profile during the year.

### Water storage change

#### Effect of sensor calibration on water storage hydrograph

**Fig. 7:**
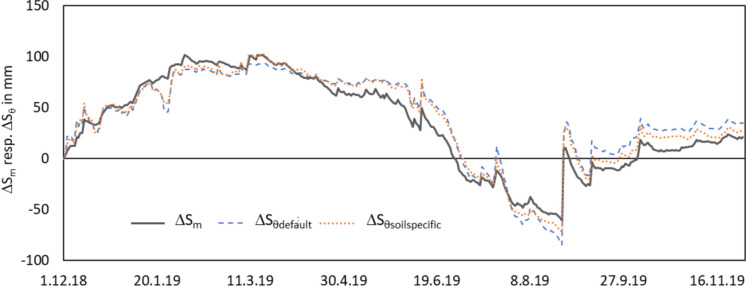
Water storage changes related to the first measured value (1st of December 2018) between the 1st of December 2018 and 30th of November 2019, actual water storage change (ΔS_m_), default water storage change (ΔS_θdef_), and soil specific water storage change (ΔS_θspec_)

Both water content-based hydrographs overestimate as well as underestimate the actual water storage change hydrograph (Fig. 7). Up to the end of the time series, both hydrographs nearly reach the value of actual water storage change hydrograph again. The deviation is only 5.8 mm for the soil specific storage change and 14.0 mm for the default storage change. During the most time (up to end of July), the differences are smaller between the water content-based hydrographs (ΔS_θdefault_ vs. ΔS_θsoil specific_) than their deviation to the actual storage change graph. Only in August, the time with the deepest groundwater levels, when the third soil horizon takes part to the storage change and the actual storage change is underestimated, the differences between default and soil specific calibrated water storage changes are bigger than the difference between actual storage change and soil specific water storage change. Increases of the water storage are referred to precipitation or inflow to the lysimeter via groundwater (sub-irrigation). Decreases of the water storage are attributed to withdrawal of water by the plants (evapotranspiration) or discharge (drainage). Both reactions are reflected by all hydrographs well. The statistical metrics (Table 3) and the quality criteria (Table 4) attest an improvement of the water storage change determination by water content measurement with the soil specific calibration.

**Table 3:** Statistical metrics of the actual water storage change (ΔS_m_), default water storage change (ΔS_θdef_), and soil specific water storage change (ΔS_θspec_)

	**WTD in cm**	**ΔS**_**m**_ ** in mm/d**	**ΔS**_θ**def**_ ** in mm/d**	**ΔS**_θ**spec**_ ** in mm/d**
Min	−126.5	−60.9	−84.4	−72.4
Mean	−52.5	32.9	38.7	37.5
Median	−56.0	29.5	40.5	39.5
Max	−2.6	102.1	93.4	102.4
Range	123.9	163.0	177.8	174.8
Stdev	35.9	45.5	44.0	44.8

**Table 4:** Quality criteria of the comparison of the actual water storage change with the default and soil specific water storage change of the period between the 1st of December 2018 and 30th of November 2019

Criteria	Default	Soil specific
R^2^	0.96	0.98
IoA	0.98	0.99
Bias in mm	−5.85	−4.61
RMSE in mm	14.51	10.82
NSE	0.90	0.94

The hydrographs of Fig. 7 show some peculiarities. While the actual water storage is only slightly underestimated up to mid of April, particularly some significant underestimations in December and end of January stands out. This was a period with air temperatures below −5 °C. But the soil temperatures stayed in the positive range. That means that the frost has not affected the measurement volume of the soil. But the electronical part in the head of the probe and possibly the air in the well of the probe were affected. In December 2018, the water contents in 20 and 30 cm decreased for a short time and raised again (Fig. 6) with effects on the calculated water storage. In the end of January, only the 10 cm depth showed this behaviour. Both indicates that the saturation of the soil has also an effect for this special behaviour of the measured values in periods with frost. In December, the water table depth was between 20 and 30 cm below surface. The first sensor of the EnviroScan probe was not completely surrounded by saturated soil and did not react to the deep temperatures. Up to end of January, the water levels have risen up to 12 cm below surface and the first sensor was completely surrounded by a saturated soil. Now only the first sensor had a reaction with a short decrease of the water content with deep temperatures and an increase of the water content with the increasing temperatures despite constant water levels. The water contents of the deeper sensors did not react to the change in air temperature.

The period with generally dropping groundwater levels from mid of April up to end of August contains both, period with overestimations of the storage changes as well as periods with underestimations. The switch between both phases is in the mid of July, when the groundwater levels fall below 90 cm under surface. This is a hint for inaccuracies in the quantification of the water storage in the third horizon. One reason is probably the bigger distance between the sensors below 60 cm depth. The two deepest sensors have a distance of 30 cm. The deepest sensors represent a thicker layer than the sensors near surface with 10 cm distance. Hence, the inaccuracies in the determination of the water storage must increase. The sensor in 60 cm is relevant for the water storage of the soil layer from 55 to 75 cm (20 cm thick), the sensor in 90 cm depth is relevant for the water storage between 75 and 105 cm (30 cm thick), and the sensor in 120 cm depth is relevant for the soil layer between 105 and 135 cm. Hence, their measured water contents were possibly applied to parts of the soil profile which were still saturated and had no change in the water storage. So, the water storage change could be overestimated. A constant distance between the sensors up to 120 cm or the depth which is always saturated could help to improve the accuracy of the measurement.

Rainfall events are reflected in the water storage hydrographs by both methods clearly. While all small events are also reproduced in the weighted hydrograph very well, they are hardly observable in the water content-based water storage changes. Possible reasons are the small measurement volume of the water content measurements compared to the whole volume of the lysimeter. Preferential flow in macro pores can also play a role (Davies et al., 2024). After larger rainfall events, the reaction of the water content-based water storage change is often stronger than the actual water storage change, as shown for events in June and August 2019 (Fig. 7).

The differences between the default water storage change and the soil specific water storage change are relatively small. The deviations of the soil specific water storage change to the actual water storage change are slightly smaller as the quality criteria show (Table 4). But the default calibration would also describe the water storage change well in the annual hydrograph under the soil conditions of the study site in the Spreewald wetland. But it is not clear that this would also be the case for thicker peat layers with a much higher organic matter content and generally higher water contents.

The second horizon of the soil profile is not really participating in the water storage change because of its thin thickness and the specific soil properties (see Sect. ‘Volumetric water content measurements’). The third horizon participates only partly to the water storage change during the summer months, i.e. when the groundwater levels are deeper than 60 cm. The main water storage change occurs in the first horizon within the upper 40 cm. Here, the plant roots extract water and the soil dries out. In the horizons below this depth, the water content changes are only small because of the interaction of soil properties, missing water extraction by plants, and capillary rise from the groundwater.

#### Effect of reference level on water storage hydrograph

**Fig. 8:**
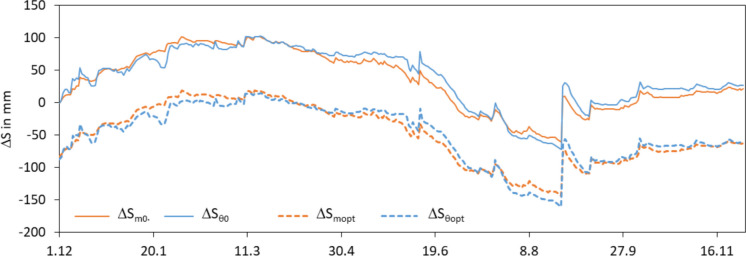
Hydrographs of the actual and soil specific water storage change between the 1st of December 2018 and 30th of November 2019. Water storage values are related the 1st of December 2018 (ΔS_m0_, ΔS_θ0_) resp. 6^th^ of April 2019 (ΔS_mopt_, ΔS_θopt_)

The reference level for the calculation of the water storage change can be selected randomly. The reference level has an effect on the absolute values of the water storage change and the difference between the actual water storage change and the soil specific water storage change but not on the relative behaviour of the hydrograph. Basis of the determinations of the storage changes according to Eqs. 4 and 6 were the first measurement values of the time series (1.12.2019). The calculated hydrographs have a good agreement of the water storage changes (Fig. 8). They were already discussed in Sect. ‘Effect of sensor calibration on water storage hydrograph’. Both hydrographs start with zero and end with a difference of 6 mm between their end values on 30th of November 2019. The default water storage change will not be considered in this section.

The optimisation of the reference level calculated the best values for the selection of the reference level of the 6th of April 2019 (Fig. 8). Table 5 shows an improved approximation of the median and mean values. Range and standard deviation are not affected by the change of the reference level.

**Fig. 9:**
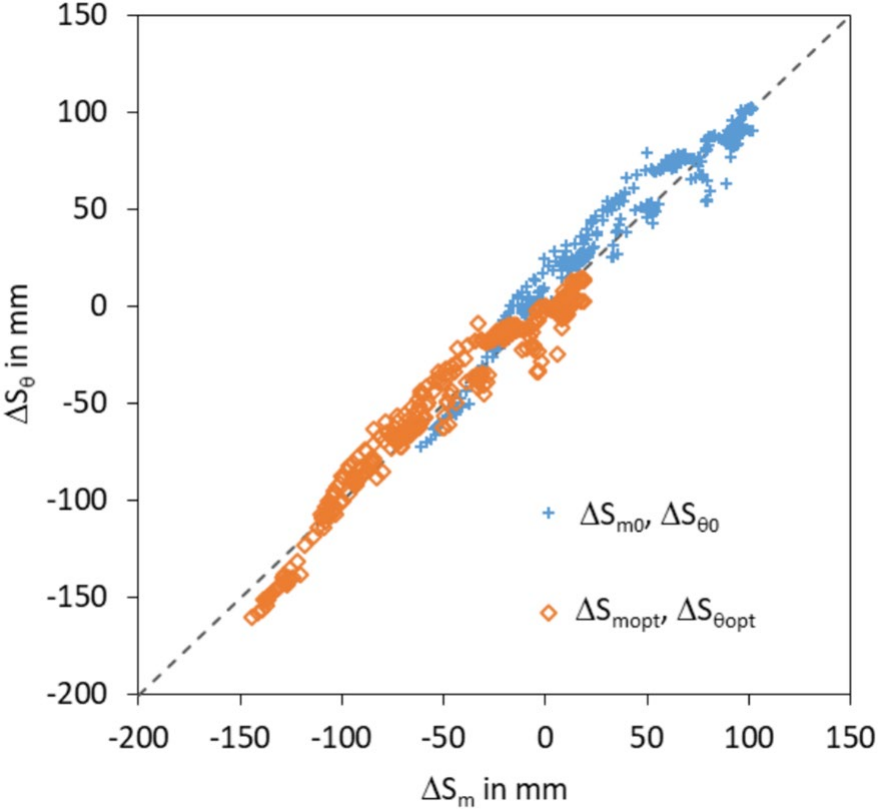
Comparison of the actual water storage change and the soil specific water storage change for the reference values first value (ΔS_m0_, ΔS_θ0_) and optimal value (ΔS_mopt_, ΔS_θopt_)

The direct comparison of the differently calculated water storage changes illustrates the effect of the modified reference level (Fig. 9). The water storage changes based on the first value of the time series show a slight overestimation of the actual water storage change by the soil specific water storage change. The shift of the reference value to the 6th of April 2019 shifts the whole values and reduces the overestimation slightly. But it does not remove the general overestimation and underestimation.

**Table 5:** Statistical metrics of the actual water storage change and soil specific water storage change referred to the first value (ΔS_m0_, ΔS_θ0_) and to the optimal value (ΔS_mopt_, ΔS_θopt_)

	**01.12.2018**		**06.04.2019**	
	ΔS_m0_	ΔS_θ0_	ΔS_mopt_	ΔS_θopt_
**Min**	−60.9	−72.4	−144.1	−160.2
**Mean**	32.9	37.5	−50.3	−50.4
**Median**	29.5	39.5	−53.7	−48.4
**Max**	102.1	102.4	18.9	14.5
**Range**	163.0	174.8	163.0	174.8
**Stdev**	45.5	44.8	45.5	44.8

The optimization of the reference level improves all quality criteria except for the correlation coefficient (Table 6). Especially, the bias was improved. The optimal reference level can change from year to year and be any point of the time series. This makes a comparability of time series from different years difficult. Under practical aspects, the selection of a fixed reference point on a distinctive point of the annual cycle makes more sense than a flexible point that minimises the differences between actual and soil specific water storage change. But the reference value should be selected every year new. Otherwise, derivations from the actual year will be taken to the next year. For example, a fixed date at the beginning of the vegetation period could be a good selection. At this time, the soil is completely saturated, the water storage in the soil does not change, and the water levels above the surface are no longer as high as it is in winter.

**Table 6:** Quality criteria of the comparison of actual water storage change and soil specific water storage change of the period between the 1st of December 2018 and 30th of November2019. The values are related to the first value (ΔS_m0_, ΔS_θ0_) and the optimal value (ΔS_mopt_, ΔS_θopt_)

Reference value	ΔS_m0_, ΔS_θ0_	ΔS_mopt_, ΔS_θopt_
Reference date	01.12.2018	06.04.2019
R^2^	0.95	0.95
Bias	−4.61	0.08
IoA	0.99	1.00
RMSE	10.82	9.78
NSE	0.94	0.95

#### Daily values of the water storage change

For determining the water storage change for any time period, (e.g. the daily storage change or the storage change in a month), the reference time to be selected comes from the time step itself. The result of both methods (Eqs. 4 and 6) is always unambiguous, and the values can be compared directly. It is a disadvantage that the single values do not illustrate the temporal development of the measured value.

**Fig. 10:**
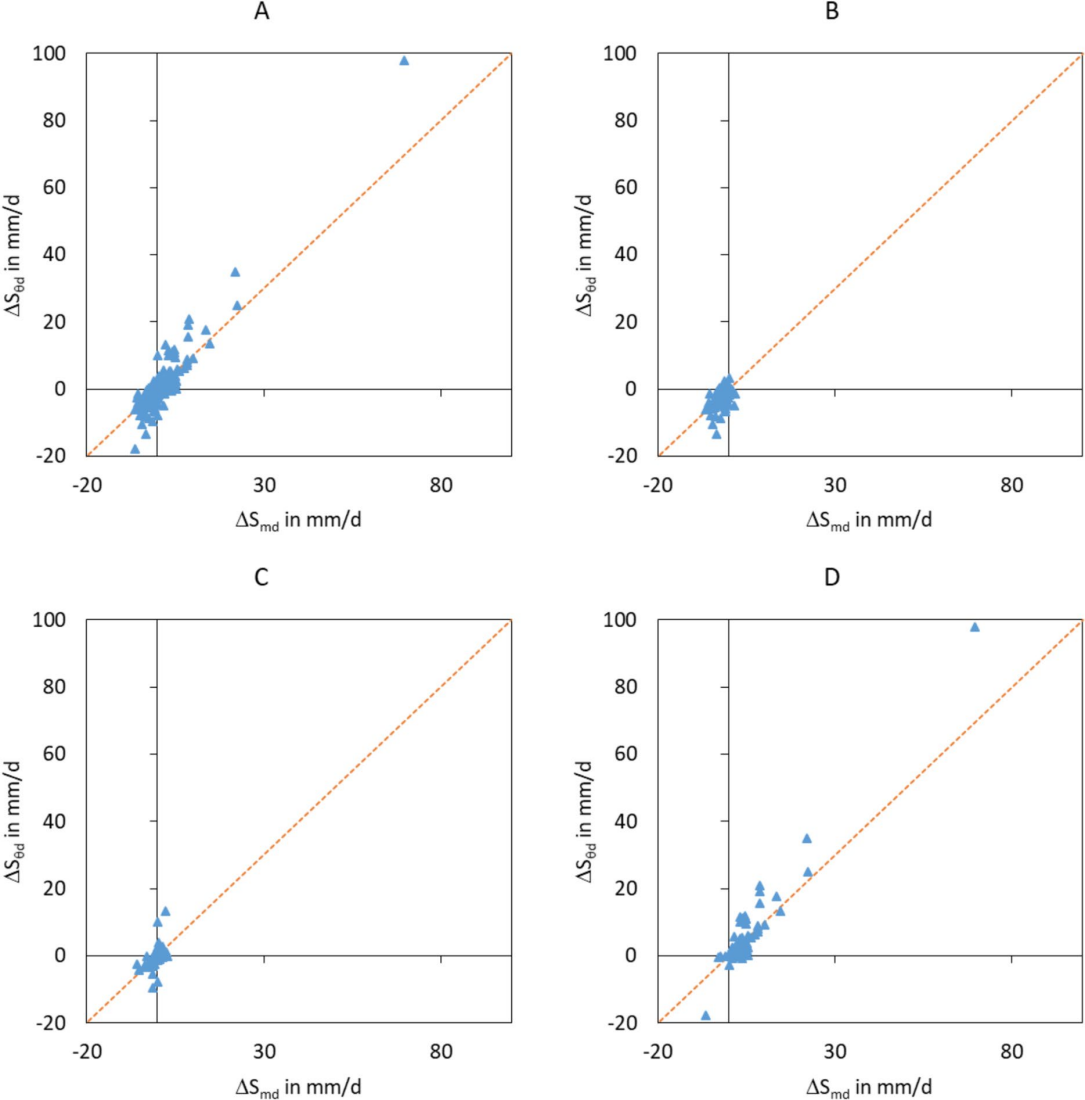
Comparison of the daily actual and soil specific water storage change (ΔS_md_, ΔS_θd_) between the 1st December of 2018 and 30th of November 2019 (A: all data of the time series, B: only data without precipitation (*P* = 0 mm/d), C: 0 < *P* < 2 mm/d, D: *P* >  = 2 mm/d)

**Fig. 11:**
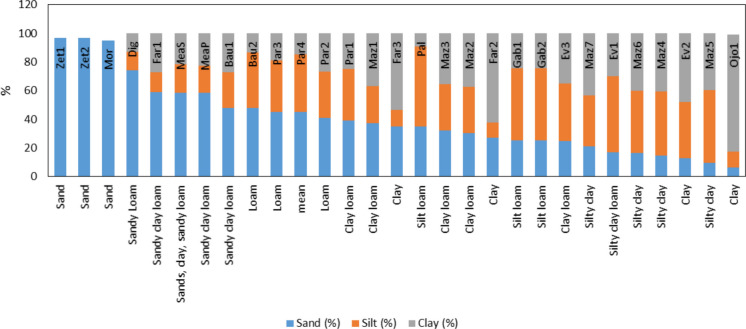
Soil textures of soils with soil specific calibration function for the EnviroScan probe in Table 9

**Fig. 12:**
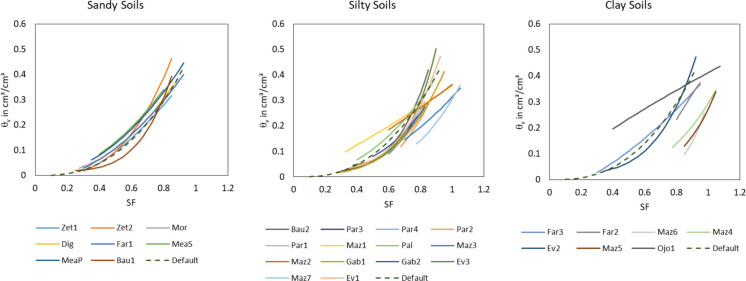
EnviroScan calibration functions of Table 9, grouped by soil texture

The comparison of daily values of differently determined water storage changes shows overestimations and underestimations of the specific soil water storages (Fig. 10A, Tables 7 and 8). These occur often related to bigger rainfall events. A possible reason is that the mass of the lysimeter directly increases with the beginning of the rainfall and the increase stops with the end of the rainfall. The water content-based storage determination can have delayed reactions because the soil moisture penetration does not occur uniformly in time and space. The water infiltrates and flows in preferential pathways and is distributed timely delayed within the whole soil profile. This can lead to timely delayed changes in the water content of the relatively small measurement volume of the profile probe sensor and affect the calculated water storage changes up to the following day. Numerical model simulations (Davies et al. (2024) suggested that macropore flow after rainfall events could also be a relevant flow process in the lysimeter.

Another reason for deviations of the soil specific water storage change is the different measurement volume. While the actual water storage change is based on the whole volume of the lysimeter monolith (2 m^3^), the soil specific water storage change is only based on upscaled water content measurements on a volume of eight sensors in 10 cm surrounding the 2 in. well of the EnviroScan probe. Soil heterogeneities in the lysimeter monolith can sophisticate the upscaled values. But this is a problem of every upscaling of measured point data to the next bigger scale (Wendroth et al. 2013), also a problem of upscaling of the lysimeter data to a larger area.

The statistic metrics of the soil specific water storage change do not really deviate in median and mean value. But minimum and maximum values and consequently the range deviate clearly from the daily actual water storage change (Table 7, all data). However, all quality criteria in Table 8 underline that the soil specific data reflect the actual data well.

In a next step, the daily water storage changes were grouped to three classes in depending on the daily precipitation sum. Data set 1 contains all days without precipitation (*P* = 0 mm/d, Fig. 10B), data set 2 all days with a precipitation sum *P* < 2 mm/d (Fig. 10C) and data set 3 all data with a precipitation sum *P* >  = 2 mm/d (Fig. 10D). The classification helps to illustrate the effect of the precipitation on quality of the water storage measurements.

**Table 7:** Statistical metrics of the daily actual and soil specific water storage change (ΔS_md_, ΔS_θd_) between the 1st of December 2018 and 30th of November 2019 for all data and classified by precipitation (*P*)

	**All data**		***P*** ** = 0 mm/d**		**0 < ** ***P*** ** < 2 mm/d**		***P*** ** > = 2 mm/d**	
	**ΔS**_**md**_	**ΔS**_θ**d**_	**ΔS**_**md**_	**ΔS**_θ**d**_	**ΔS**_**md**_	**ΔS**_θ**d**_	**ΔS**_**md**_	**ΔS**_θ**d**_
**Number**	356	352	213	212	73	72	70	68
**Min**	−6.4	−17.8	−6.3	−13.4	−5.9	−9.6	−6.4	−17.8
**Mean**	0.0	0.0	−1.6	−1.9	−0.3	−0.2	5.0	6.1
**Median**	−0.7	−0.6	−1.3	−1.4	0.0	−0.2	3.2	2.4
**Max**	69.5	97.9	1.9	3.2	2.7	13.3	69.5	97.9
**Range**	75.9	115.7	8.2	16.6	8.6	22.9	75.9	115.7
**Stdev**	4.9	6.9	1.4	2.1	1.6	2.9	9.0	13.4

**Table 8:** Quality criteria of the comparison of the daily actual and soil specific water storage change (ΔS_md_, ΔS_θd_) between the 1st of December 2018 and 30th of November 2019 for all data and classified by precipitation (*P*)

	**All days**	***P*** ** = 0 mm/d**	**0 < ** ***P*** ** < 2 mm/d**	***P*** ** > = 2 mm/d**
R^2^	0.87	0.31	0.23	0.93
Bias	0.03	0.36	−0.03	−0.89
IoA	0.94	0.88	0.59	0.96
RMSE	2.95	1.82	2.56	5.23
NSE	0.64	−0.65	−1.69	0.67

On days without precipitation or few precipitation (< 2 mm/d), the daily water storage change is mostly negative because more water is extracted from the water storage by evapotranspiration than the inflow from groundwater or precipitation can supply. The actual water storage changes are slightly overestimated on days without precipitation (Fig. 10B). The relatively small values for the storage change could be the main reason for this, because we are at the limit of the measuring accuracy of the water content measurement. Analysing longer periods of time could reduce this problem. The RSME values of data set 2 are smaller than the RSME of the complete data set (Table 8). But also, the quality criteria R^2^, IoA, and NSE are smaller.

Deviations of the soil specific water storage change from the actual water storage change occur especially on days that are following days with more precipitation (*P* >  = 2 mm/d) because of drainage. It explains the overestimation of the minimum values in Table 7. Again, the different measurement volume and possible macropore flow paths should be the reason for the deviations. Days without precipitation could also be affected by this effect if they are a following a day after a day with precipitation.

Most of the quality criteria (bias, IoA, RSME) worsen on days with precipitation (Table 8). This is primarily due to the aforementioned problems of different measurement volumes. The criteria R^2^ and NSE improve slightly with increasing precipitation because of generally bigger storage change values with hence smaller problems with the sensor accuracy and the bigger range of the values. The classification of the storage change values by the precipitation sums illustrates some important problems of the water content-based data which the user should be aware. It also shows that all data can be used if their specific problems are sufficiently considered.

#### Restrictions and limitations

There are some limitations for the determination of water storage changes with soil moisture measurements on shallow groundwater sites. Frequently, the water levels are above the surface here. Especially, the local depressions at these sites can be temporally flooded. Such conditions are typical for natural wetlands and are in the focus of many water management measures for the improvement of water retention in landscapes. Inundation conditions can also be simulated with groundwater lysimeters (Dietrich et al., 2016) that can measure the water storage change of whole storage volume, including the part above the surface, with a weighing system. Under flooded conditions, the water storage change cannot be determined by water content measurements. It does not play a role how many sensors are installed in the profile, if there are heterogeneities in the soil profile or which type of sensors are used. The water content-based method can only be applied if the water levels are below surface, and an unsaturated zone exists in the soil profile where the water content is changing. If the water level is only a few centimetres below surface, the changes in the water contents can also be small if the capillary fringe reaches the surface and if the water retention function of the soil has a steep slope near saturation.

The quality of the water storage measurement with profile probes is also affected by the length of the selected time discretisation. Lysimeter measurements are exact for each time step. On the one hand, the water storage changes estimated by water content measurements reflect its temporal behaviour as shown by the cumulative curves (Fig. 7). On the other hand, the analysis of shorter time periods, such as individual daily values, may contain greater inaccuracies (Fig. 10, Table 8). Specific processes, such as macropore flow, or soil heterogeneities can become more important at shorter time scales. The comparatively small measurement volume is also becoming more relevant. The same applies to larger individual events, such as heavy rainfall. For longer time steps, the effects of these special processes as well as the effects of heterogeneities become less relevant. These limitations of the EnvironScan probe have a relevance on all site conditions independent of groundwater conditions and soil types. This is relevant for scientific investigations. But for the evaluation of the effects of water management measures like the improvement of the water retention in the landscape by rewetting, a relatively small accuracy has a lower importance. In this context, it is more important to consider longer periods of time. Here, the estimation of changes in water storage using profile probes can be effective. However, the sensors of the profile probes should always be calibrated for the specific soil type, especially in soils with a high organic matter content. At sites with shallow groundwater conditions, the spatial discretisation of profile probe sensors across the soil profile in the range of the expected groundwater fluctuations should be relatively dense, especially if soil horizons vary along the profile depth. Larger sensor distances always require more interpolation of moisture contents between sensor depths, which may lead to additional inaccuracies.

Capacitive water content sensors, like the used EnviroScan probe, are frequently temperature-affected (Evett et al., 2006; Paltineanu & Starr, 1997). The probe-measured values tend to overestimate the soil water contents depending on the temperature; overestimation increases with temperature. Hence, the calculated water storage changes are also overestimated when the water storage rises depending on the temperature and the fluctuation of the groundwater levels. Temperature-dependency affect data also when the water storage decreases, then storage change is underestimated since the water level drops or the soil is drying. This can partly explain the underestimation in the summer months (Fig. 7). The temperature problem is less important for the comparison of the daily water storage changes because the temperature changes in the soil are relatively slow processes. They are more relevant for the hydrograph of the water storage change.

## Conclusions

The objective of the present study was the evaluation of the suitability of a capacitive soil moisture profile probe for the estimation of the soil water storage change on a shallow groundwater site. Continuous measurements of volumetric water contents with profile probes could sufficiently well describe water storage changes of shallow groundwater sites as long as the water levels are below the surface. If the soil is saturated up to the surface or the surface is inundated, water storage changes have to be combined with models and have to consider the topography of the site.

The comparison of the volumetric water contents with default and the soil specific calibration underlines the necessity of a soil specific calibration of the capacitive EnviroScan probe for this site. The default calibration only provides sufficiently accurate water contents for soils that correspond to the soil on which the default calibration is based. Especially, the water contents of the soil with a higher organic matter content are underestimated. However, the quantification of the water storage changes of the lysimeter was not as much affected by the soil specific calibration as expected. The overestimation and underestimation of the actual water storage change by both sensors’ water content-based water storage changes was larger as the improvement due to the soil specific calibration. Furthermore, results demonstrated the important effect of soil physical properties of specific low-permeable and organic matter-containing soil horizons.

## Data Availability

The data are published in the data repository BonaRes, 10.4228/zalf-2cy6-gk16

## Appendix

Literature review of existing calibration functions of the EnviroScan probe.

**Table 9:** Summary of soil properties (C_org_, **ρ**_**b**_**)**, parameter of soil specific calibration functions (A, B, C), quality criteria (R^2^, RSME), range of measurement of volumetric water contents (θ_min_, θ_max_) and scaled frequencies (SF_min_, SF_max_)

**Reference**	**Abbreviation**	**Soil**	**C**_**org**_	**ρ**_**b**_	**A**	**B**	**C**	***R***^**2**^	**RSME**	**θ**_**min**_	**θ**_**max**_	**SF**_**min**_	**SF**_**max**_
			(%)	(g/cm3)					**(cm**^**3**^**/cm**^**3**^**)**	(cm3/cm3)	(cm3/cm3)		
Sentek (2011)	Default	Sands, loams and clay loams	-	-	0.5299	2.5775	0	0.9737	-	0.04	0.35	0.37	0.86
Mead et al. (1995) in Sentek (2011)	MeaS	Sand, clay, sandy loam	-	-	0.5312	2.0022	0	0.973	-	0.05	0.30	0.39	0.80
Paraskevas et al. (2012)	Par1	Clay loam	-	1.08	0.5501	3.2449	0	0.868	-	0.09	0.30	0.57	0.83
	Par2	Loam	-	1.30	0.5301	3.7974	0	0.949	-	0.11	0.30	0.67	0.86
	Par3	Loam	-	1.11	0.6035	3.7073	0	0.992	-	0.08	0.24	0.58	0.78
	Par4	Avg of Par1–Par3	-	1.16	0.5382	3.4421	0	0.89	-	0.08	0.29	0.58	0.84
Paltineanu and Starr (1997)	Pal	Silt loam	0.8	1.41	0.4900	2.1674	0	0.992	0.009	0.07	0.37	0.39	0.89
Mead et al. in Paltineanu and Starr (1997)	MeaP	Sandy clay loam	-	1.28	0.5220	2.0460	0	0.932	0.031	0.04	0.45	0.33	0.94
Dighton and Dillon (1993, unpublished data) in Paltineanu and Starr (1997)	Dig	Sandy loam	-	1.34	0.5000	2.2310	0	0.979	0.016	0.04	0.42	0.35	0.90
Morgan et al. (1999)	Mor	Sand	-	-	0.4514	2.1211	0	0.831	-	0.02	0.08	0.27	0.43
Baumhardt et al. (2000)	Bau1	Sandy clay loam	-	-	0.7520	4.2600	0.017	0.955	0.032	0.02	0.40	0.23	0.86
	Bau2	Loam	-	-	0.7980	4.2500	0.02	0.993	0.014	0.03	0.39	0.28	0.86
Fares et al. (2004)	Far1	Sandy clay loam	0.35	1.40	0.4867	1.9480	−0.018	0.94	0.027	0.04	0.35	0.30	0.93
	Far2	Clay	0.07	1.63	1.6050	0.5520	−1.186	0.89	0.017	0.18	0.41	0.78	0.95
	Far3	Clay	0.14	1.58	0.4400	1.5490	−0.04	0.88	0.029	0.04	0.41	0.30	0.95
Evett et al. (2006)	Ev1	Silty clay loam	-	1.42	0.6050	3.8120	0.024	0.993	0.022	0.05	0.43	0.35	0.93
	Ev2	Clay	-	1.45	0.6050	3.8120	0.024	0.993	0.022	0.06	0.47	0.35	0.93
	Ev3	Clay loam	-	1.41	0.7810	4.9810	0.041	0.996	0.018	0.04	0.48	0.36	0.90
Mazahrih et al. (2008)	Maz1	Clay loam	-	1.24	0.3576	1.1440	0	0.931	0.025	0.09	0.36	0.31	1.02
	Maz2	Clay loam	-	1.24	0.3626	1.3100	0	0.913	0.021	0.18	0.36	0.58	1.00
	Maz3	Clay loam	-	1.22	0.3133	2.1310	0	0.916	0.025	0.14	0.36	0.68	1.05
	Maz4	Silty clay	-	1.18	0.2932	3.3390	0	0.824	0.039	0.13	0.36	0.77	1.05
	Maz5	Silty clay	-	1.14	0.2709	4.5660	0	0.858	0.031	0.13	0.33	0.85	1.05
	Maz6	Silty clay	-	1.20	0.3058	7.0790	0	0.511	0.051	0.08	0.28	0.83	0.99
	Maz7	Silty clay	-	1.15	0.3047	3.3900	0	0.635	0.039	0.13	0.36	0.77	1.05
Gabriel et al. (2010)	Gab1	Silt loam	0.018	1.30	0.4780	3.3030	0.01	0.96	0.027	0.02	0.45	0.27	0.95
	Gab2	Silt loam	0.018	1.44	0.4440	3.0490	0.027	0.92	0.024	0.06	0.30	0.49	0.84
Ojo et al. (2015)	Ojo1	Clay	7.8	-	0.4121	0.8088	0	0.71	0.04	0.24	0.46	0.40	1.08
Zettl et al. (2015)	Zet1	Sand	-	1.76	0.6223	1.5803	−0.1978	0.94	-	0.03	0.34	0.30	0.86
	Zet2	Sand	-	1.57	2.9773	2.7522	0.0467	0.974	-	0.03	0.34	0.30	0.86
